# Bladder perforation during transurethral resection of bladder tumour is not a result of a deficient structure of the bladder wall

**DOI:** 10.1186/s12957-020-01992-8

**Published:** 2020-08-19

**Authors:** Sławomir Poletajew, Tomasz Ilczuk, Wojciech Krajewski, Grzegorz Niemczyk, Agata Cyran, Łukasz Białek, Piotr Radziszewski, Barbara Górnicka, Piotr Kryst

**Affiliations:** 1 Second Department of Urology, Centre of Postgraduate Medical Education, 80 Cegłowska St., 00809 Warsaw, Poland; 2 Department of Pathology, Medical University of Warsaw, Warsaw, Poland; 3 Department of Urology and Oncological Urology, Wrocław Medical University, Wrocław, Poland; 4 Department of General, Oncological and Functional Urology, Medical University of Warsaw, Warsaw, Poland; 5 First Department of Urology, Centre of Postgraduate Medical Education, Warsaw, Poland

**Keywords:** Electron microtomography, Intraoperative complications, Transurethral resection, Urinary bladder, Ultrastructure

## Abstract

**Background:**

Transurethral resection of the bladder tumour (TUR) is associated with a risk of bladder perforation. The underlying mechanisms and risk factors are not fully understood. The aim of this study was to determine if the bladder wall structure affects the risk of bladder perforation during TUR.

**Methods:**

Fifteen patients who underwent TUR complicated by a bladder perforation (group 1) and fifteen matched controls who underwent uncomplicated TUR (group 2) were retrospectively enrolled in this morphological analysis. Surgical specimens were collected from all participating patients to describe the quality and architecture of urothelium and bladder submucosa. Immunohistochemical studies were performed with primary mouse anti-human E-cadherin, beta-catenin, type IV collagen, cytokeratin 20 and epithelial membrane antigen antibodies. The intensity of the immunohistochemical reaction was assessed using an immunoreactive score (IRS). Ultrastructural examinations were performed by transmission electron microscopy. The microscopic assessment was focused on the intensity of fibrosis in the bladder submucosa and the presence of degenerative changes in the urothelium.

**Results:**

Patients’ age, sex distribution, tumour diameters, surgeon experience or cancer stage did not differ between study groups. The immunohistochemical analysis did not reveal statistically significant differences between group 1 and group 2. From a clinical point of view, ultrastructural analysis by electron microscopy showed a higher rate of severe fibrosis in group 1 (63.6% vs. 38.5%), with no differences in the rate and degree of urothelial changes. However, these differences were not statistically significant (*p* = 0.32).

**Conclusions:**

Bladder perforation during TUR is not a result of a deficient structure of the bladder wall. Based on available evidence, the surgical technique seems to play the most important role in its prevention.

## Introduction

Bladder cancer is the most common urinary tract neoplasm, while transurethral resection of a bladder tumour (TUR) is one of the most commonly performed urological procedures [1, 2]. Among possible complications, bladder perforation is both a frequent and significant one, as it has direct surgical and oncological consequences [3]. Data on the risk factors for bladder perforation at the time of TUR are scarce and limited mainly to the issue of the surgical experience of the urologist [4].

- The bladder is by far the urological organ most commonly affected by iatrogenic trauma, mainly as a complication of gynaecological or urological procedures [5, 6]. Bladder perforations are categorised as intraperitoneal, extraperitoneal or combined, and these categories indirectly indicate further management [7, 8]. Apart from the surgeon’s experience, the risk of bladder perforation during TUR increases with tumour size, location in the bladder dome, patient age, and history of previous bladder surgery [9, 10]. The vast majority of bladder perforations at the time of TUR are extraperitoneal and only 0.2–0.6% of patients require surgical intervention [9–11]. However, the affected patients need prolonged bladder catheterisation, antibiotic therapy and follow-up with control imaging studies [5, 12].
  Based on subjective clinical observations, we hypothesised that bladder perforation does not result only from surgical technique, but also from the abnormal bladder wall structure. This would explain why even experienced urologists can perforate the bladder during TUR and why, during the surgery, experienced resectionists can subjectively predict a higher risk of perforation due to reduced bladder wall compliance.

The aim of this study was to determine if the bladder wall structure affects the risk of bladder perforation during TUR.

## Material and methods

This was a retrospective clinical study based on a prospectively collected database of consecutive patients undergoing TUR for bladder tumours from January 2015 to December 2017 in three academic institutions.

### Patients

Fifteen consecutive patients who underwent TUR complicated by a bladder perforation (group 1) and fifteen matched controls who underwent uncomplicated TUR (group 2) were retrospectively enrolled in this morphological analysis. Bladder perforation was diagnosed based on endoscopic images. Confirmatory retrograde urethrocystography was performed in 11 cases (73.3%) at the surgeon’s discretion. Additional diagnostic procedures were avoided in evident cases. Patients in group 2 were identified from our institutional database after cognitive matching based on gender, age, bladder cancer history (primary vs. recurrent tumour), tumour size and pathological stage, experience of the surgeon (resident vs. certified urologist).

All patients gave signed written consent to participate in the study. The approval of the institutional review board was waived for this retrospective and non-interventional study, according to local regulations.

### Specimen handling

Surgical specimens were collected from all participating patients at the time of TUR as a part of routine clinical care. After completion of the surgery, the tissues were fixed in formalin by immersion to be finally dehydrated and embedded in paraffin blocks. After initiation of this study, archival microscopic slides of all patients’ tumours, stained with H&E, were re-evaluated by an experienced uropathologist to choose a paraffin block containing the most representative image of urothelial cells and bladder submucosa with no cancer for final analysis.

Immunohistochemical and ultrastructural analyses were used to determine the quality and architecture of urothelium and bladder submucosa. Particular interest was paid to the degenerative or reactive changes and fibrotic processes.

### Immunohistochemical examination

Paraffin blocks were serially cut into 3-μm slices with a microtome for immunohistochemical staining. Antigen retrieval was performed by a 20-min thermal incubation in Target Retrieval Solution (Dako, Denmark) in all cases. Staining was performed in an automatic station (Dako, Denmark).

The choice of antibodies was based on their ability to identify degenerative or reactive changes and fibrotic processes within urothelium and bladder submucosa. The following primary antibodies were used: mouse anti-human E-cadherin (clone NCH38, Dako IS059, Denmark), mouse anti-human beta-catenin (clone beta-catenin 1, Dako IS702, Denmark), mouse anti-human type IV collagen (clone CIV22, Dako M0785, Denmark), mouse anti-human cytokeratin 20 (clone KS20.8, Dako IS777, Denmark), and mouse anti-human epithelial membrane antigen (clone E29, Dako I629, Denmark). Only ready to use, autostainer-dedicated reagents were used.

For an objective assessment of the immunohistochemical reaction intensities, we adopted the immunoreactivity score (IRS) scale designed by Remmele and Stagner [13]. This is a semi-quantitative scale incorporating the percentage of positive cells and staining intensity in five visual fields of the light microscope at ×200 magnification. The final IRS is a product of the percentage of positive cells (score of 0, no cells with positive reaction; 1, ≤ 10% cells with positive reaction; 2, 11 to 50% cells with positive reaction; 3, 51 to 80% cells with positive reaction; 4, > 80% cells with positive reaction) and staining intensity (0, no colour reaction; 1, poor colour reaction; 2, moderate colour reaction; 3, intensive colour reaction). IRS values can range from 0 to 12 (0–2, poor reaction; 3–5, moderate reaction; 6–12, intense reaction).

### Ultrastructural examination

Ultrastructural examination was performed on material from paraffin blocks, which were deparaffinized, dehydrated, fixed in osmium tetroxide, and embedded in an epoxy resin. The polymerization of the resin was carried out at increasing temperatures: 37 °C and 45 °C on the first day, and 60 °C in the next 2 days. Sections were then applied to a metal mesh of a 3-mm diameter and contrasted with heavy metal salts, uranyl acetate, and lead citrate. Finally, the material was assessed using transmission electron microscopy.

The microscopic assessment was focused on two issues: (1) the intensity of fibrosis in the bladder submucosa and (2) the presence of degenerative changes in the urothelium (including the loss of intercellular adhesion and junctions, the loss or fragmentation of nuclei, the increase of extracellular matrix, presence of leukocytes, and presence of areas of increased electron density of unknown character). To avoid the descriptive presentation of the results, subjective classifications using scores of 1–4 for both endpoints were adopted (0, no fibrosis in the submucosa or no changes in the urothelium; 1, mild fibrosis in the submucosa or mild changes in the urothelium; 2, moderate fibrosis in the submucosa or moderate changes in the urothelium; 3, severe fibrosis in the submucosa or severe changes in the urothelium).

### Statistical analysis

The clinical data are presented as absolute or mean values. Results of the immunohistochemical analysis are presented as mean IRS values, while results of structural analysis are presented by description using the adopted scale. To compare the two study groups, an unpaired *t* test or Mann-Whitney *U* test was used for quantitative variables and Pearson’s chi-square test for qualitative variables. A two-sided *p* value of < 0.05 was considered statistically significant.

## Results

The final per protocol analysis was based on 24 patients, including 11 from group 1 and 13 from group 2. Six patients were excluded from the study due to unsatisfactory images of urothelial cells and/or bladder submucosa (low quality of the tissue, artefacts, cancer cells in all slides, no submucosa). The mean age of the cohort was 73.5 years, and the male to female ratio was 13:11. The basic demographic and oncological characteristics of the patients in per protocol analysis are presented in Table 1. Group 1 did not differ from group 2 in the most significant clinical parameters.

**Table 1:** Basic oncological and surgical characteristics of the study population (per protocol analysis)

		Group 1 (perforation)	Group 2 (no perforation)	*P* value (group 1 vs. group 2)	Total
Number of patients		11	13	n.a.	24
Men		7	6		11
Women		4	7	0.39 *	13
Mean age of patients (years)		74.8	72.3	0.32 **	73.5
% of recurrent tumours		45.5	38.5	0.73 *	41.7
Mean recurrence rate (for recurrent tumours)		0.58/year	1.18/year	0.17 ***	0.92/year
Mean tumour diameter (centimetres)		2.25	1.68	0.45 ***	1.94
% of operations performed by residents in training		45.5	53.8	0.22 *	50.0
Stage of bladder cancer	Ta	8	13	0.13 *	21
	Tis	0	0		0
	T1	2	0		2
	≥ T2	1	0		1
Cancer grade	Low grade	6	11	0.11 *	17
	High grade	5	2		7

Stage of bladder cancer (according to TNM classification)

*Ta* non-invasive papillary carcinoma, *Tis* carcinoma in situ, *T1* tumour invades subepithelial connective tissue, *T2* tumour invades the muscle

*Pearson’s chi-square test

**unpaired *t* test

***Mann-Whitney *U* test

Table 2 presents surgical outcomes and in-hospital complications. Operative time and length of hospitalization did not differ between study groups. There was one case of bleeding requiring re-intervention and blood transfusion in group 1 and one case of urinary retention in group 2. Moreover, two patients from group 1 underwent laparotomy due to retroperitoneal bleeding or peritonitis. In total, three patients from group 1 needed re-interventions. No deaths occurred.

**Table 2:** Surgical outcomes and in-hospital complications

		Group 1 (perforation)	Group 2 (no perforation)	*p* value (group 1 vs. group 2)
Median TUR operative time (minutes)		35	20	0.13 *
Mean length of postoperative hospital stay (days)		1.5	1.2	0.34 *
Complications	Urinary retention	0%	7.7% (*n* = 1)	0.35 **
	Postoperative bleeding requiring blood transfusion and re-intervention	9.1% (*n* = 1)	0%	0.27 **
	Laparotomy for bladder perforation	18.2% (*n* = 2)	0%	0.11 **
	Clavien-Dindo grades III–IV	27.2% (*n* = 3)	0%	0.04 **
	Clavien-Dindo grade V	0%	0%	1.00 **

*Unpaired *t* test

**Pearson’s chi-square test

**Fig. 1:**
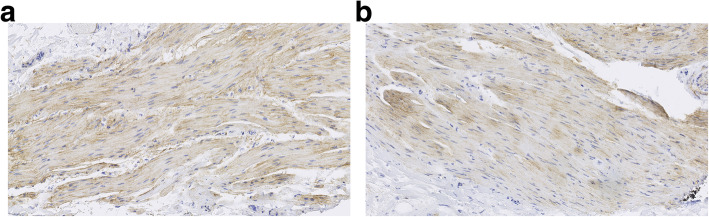
**a**, **b** Examples of microscopic images showing the expression of type IV collagen by immunohistochemistry (light microscope, ×100 magnification). Note slightly higher expression in a patient from the experimental group (**a**) as compared to a patient from the control group (**b**)

The immunohistochemical analysis did not reveal statistically significant differences between study groups, but all IRS values were higher in group 1, especially for type IV collagen and B-catenin. Detailed IRS results are presented in Table 3. Figure 1a, b presents microscopic images of the immunohistochemical expression of type IV collagen.

**Table 3:** Results of immunohistochemical analysis

			Group 1 (perforation)	Group 2 (no perforation)	*P* value (group 1 vs. group 2) *
Type IV collagen	Mean IRS score		3.24	2.20	0.49
	Percentage of positive cells	0	0%	1.8%	
		1	26.9%	54.5%	
		2	61.6%	38.2%	
		3	11.5%	5.5%	
		4	0%	0%	
	Staining intensity	0	0%	1.8%	
		1	53.8%	61.8%	
		2	38.5%	29.1%	
		3	7.7%	7.3%	
Cytokeratin 20	Mean IRS score		8.16	7.18	0.91
	Percentage of positive cells	0	0%	10.9%	
		1	19.2%	20.0%	
		2	3.8%	5.4%	
		3	61.6%	27.3%	
		4	15.4%	36.4%	
	Staining intensity	0	0%	10.9%	
		1	15.4%	9.1%	
		2	7.7%	20.0%	
		3	76.9%	60.0%	
Epithelial membrane antigen	Mean IRS score		2.84	2.11	0.41
	Percentage of positive cells	0	0%	25.5%	
		1	26.9%	32.7%	
		2	50.0%	34.5%	
		3	23.1%	7.3%	
		4	0%	0%	
	Staining intensity	0	0%	25.5%	
		1	61.5%	40.0%	
		2	38.5%	21.8%	
		3	0%	12.7%	
E-cadherin	Mean IRS score		10.64	10.45	0.79
	Percentage of positive cells	0	0%	0%	
		1	0%	0%	
		2	3.8%	0%	
		3	19.2%	45.5%	
		4	77.0%	54.5%	
	Staining intensity	0	0%	0%	
		1	0%	0%	
		2	15.4%	5.5%	
		3	84.6%	94.5%	
B-catenin	Mean IRS score		5.92	3.85	0.82
	Percentage of positive cells	0	7.7%	5.4%	
		1	23.1%	16.4%	
		2	15.4%	56.4%	
		3	46.1%	14.5%	
		4	7.7%	7.3%	
	Staining intensity	0	7.7%	5.5%	
		1	23.1%	32.7%	
		2	11.5%	50.9%	
		3	57.7%	10.9%	

Percentage of positive cells: score of 0, no cells with positive reaction; 1, ≤ 10% cells with positive reaction; 2, 11 to 50% cells with positive reaction; 3, 51 to 80% cells with positive reaction; 4, > 80% cells with positive reaction

Staining intensity: 0, no colour reaction; 1, poor colour reaction; 2, moderate colour reaction; 3, intensive colour reaction

*Pearson’s chi-square test

**Fig. 2:**
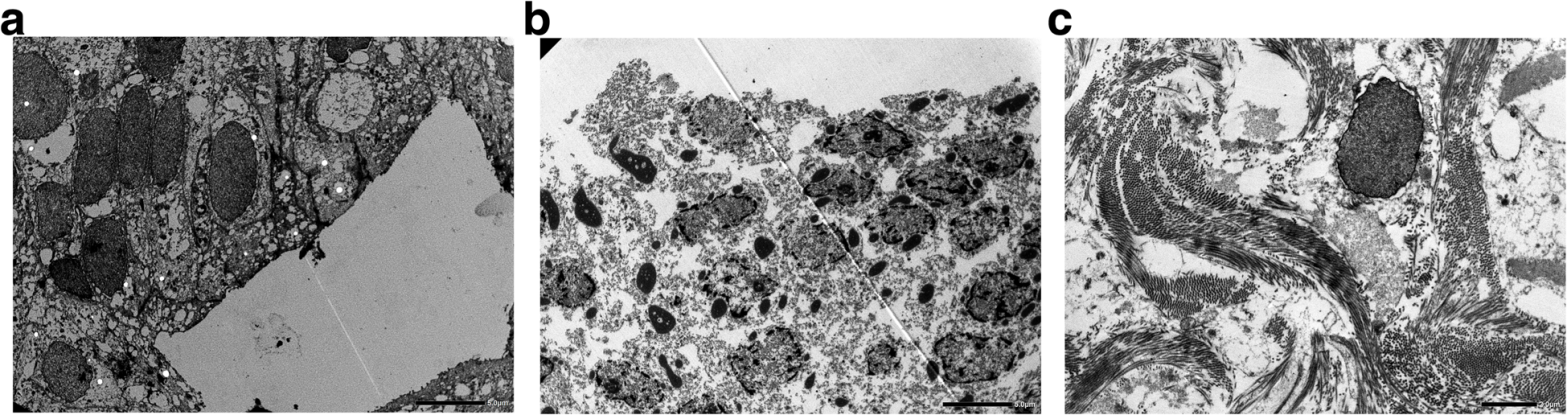
**a**–**c** Examples of electron microscopy images obtained during the study. **a** Normal bladder urothelium (×1500 magnification), **b** severe structural changes in urothelium (×1500 magnification; note ), and **c** severe fibrosis in bladder submucosa (×3000 magnification; note)

Ultrastructural analysis by electron microscopy showed a higher rate of severe fibrosis in group 1 (63.6% vs. 38.5%) with a simultaneous higher rate of moderate fibrosis in group 2 (18.2% vs. 46.1%) and no differences in the rate and degree of urothelial changes. All differences were not statistically significant. The results of the ultrastructural analysis are presented in Table 4. Figure 2a–c presents examples of electron microscopy images obtained during the study.

**Table 4:** Results of ultrastructural analysis by electron microscopy

		Group 1 (perforation)	Group 2 (no perforation)	*p* value (group 1 vs. group 2) *
Intensity of fibrosis in bladder submucosa	No fibrosis	9.1%	0%	0.32
	Mild fibrosis	9.1%	15.4%	
	Moderate fibrosis	18.2%	46.1%	
	Severe fibrosis	63.6%	38.5%	
Presence of degenerative changes in the urothelium	No changes	9.1%	7.7%	0.99
	Mild changes	0%	0%	
	Moderate changes	54.5%	53.8%	
	Severe changes	36.4%	38.5%	

*Pearson’s chi-square test

## Discussion

We performed a morphological study to determine whether structural changes within bladder mucosa and submucosa could be a cause of bladder perforation. Despite our hypothesis, we were unable to show significant differences in bladder wall morphology in patients undergoing TUR complicated by bladder perforation. The expression of selected proteins, as well as the intensity of both fibrosis within the bladder submucosa and degenerative changes within the urothelium, did not differ from those seen in uncomplicated cases.

Bladder perforation at the time of TUR in bladder cancer patients is a serious surgical complication. First, it has important surgical consequences, as it may require immediate laparotomy and vesicorraphy or at least prolonged bladder and abdominal drainage [5]. Second, it has significant oncological consequences, as bladder perforation is an absolute contraindication to intravesical chemotherapy and there is a risk of cancer-cell spillage into the perivesical region or peritoneal cavity [3, 14, 15]. In general, the incidence of bladder perforation at the time of TUR is estimated to be 0.5–8% [11, 16–20]. However, radiological signs of perforation are present in as many as 58% asymptomatic patients undergoing TUR [21].

In the past, numerous research groups have described the association between surgical experience and the risk of complications of TUR. The resident operator was established to be a risk factor for several complications, including bladder perforation [10, 20]. However, neither close supervision of a resident nor deep experience of a certified urologist eliminates this risk [10, 20]. Recently, it was shown that the resident surgeon and the presence of muscle in a specimen are independently associated with an over the three-fold higher risk of bladder perforation [4]. In view of our present results and others’ published data, the surgical technique clearly seems to play a key role in preventing bladder perforation at the time of TUR. However, a study on the learning curve for TUR showed that the risk of bladder perforation does not diminish during urological residency training. At the same time, the overall risk of TUR complication decreases after 128 procedures are performed and the best outcomes may be seen after 172 procedures [22].

The relation between the risk of bladder perforation and bladder wall structure has never been tested. However, the proper interpretation of our study results requires certain background information regarding bladder morphology. First, as a functional study by Volikova et al. has determined, bladder wall morphology is not universal. The detrusor muscle is thicker and better vascularized in men than women [23]. Second, bladder wall thickness increases with age and in the course of several lower urinary tract pathologies, including benign prostate hyperplasia, overactive bladder, and others [24, 25]. For these reasons, the risk of bladder perforation might vary between individuals. Third, the mechanical properties and microstructure of the urinary bladder wall are heterogeneous across the organ [26]. This can explain differences in the rates of bladder perforation at different locations [14]. Finally, the bladder submucosa is almost avascular [27]. This potentially increases the risk of fibrosis and hence reduction of bladder wall compliance.

Our study presents a new clinical insight, based on reproducible analysis of immunohistochemical and ultrastructural characteristics of bladder mucosa and submucosa in a representative group of patients. Yet, it has some limitations. First, all analyses were performed on archival paraffin blocks, with formalin used for the primary fixation of surgical specimens. These two facts influence the quality of the analysed tissue and it might be suspected that some of the observed phenomena were associated with tissue processing, which—in a prospective study—could be optimised for immunohistochemistry and electron microscopy. Second, the pathologists assessing microscopic images were not blinded, being aware of the study hypothesis and each patient’s clinical data. Interestingly, in both study groups, at least moderate fibrosis and at least moderate degenerative changes were noted in the clear majority of cases. The clinical meaning of this finding in relation to disease pathogenesis or surgical outcomes is unknown. Finally, the rate of high-grade tumours was higher in study group 1 as compared to group 2; however, this difference did not reach statistical significance.

In conclusion, bladder perforation during TUR is not a result of a deficient structure of the bladder wall. Based on available evidence, the surgical technique seems to play the most important role in its prevention.

## Data Availability

The datasets used and/or analysed during the current study are available from the corresponding author on reasonable request.

## Abbreviations

IRS: Immunoreactivity score
TUR: Transurethral resection of a bladder tumor

## References

[1] Svatek RS, Hollenbeck BK, Holmang S (2014). The economics of bladder cancer: costs and considerations of caring for this disease. Eur Urol.

[2] Lee F, Patel HR, Emberton M (2002). The ‘top 10’ urological procedures: a study of hospital episodes statistics 1998-99. BJU Int.

[3] Babjuk M, Bohle A, Burger M (2017). EAU guidelines on non-muscle-invasive urothelial carcinoma of the bladder: update 2016. Eur Urol.

[4] Poletajew S, Krajewski W, Gajewska D, et al. Prediction of the risk of surgical complications in patients undergoing monopolar transurethral resection of bladder tumour – a prospective multicentre observational study. Arch Med Sci. 2019. 10.5114/aoms.2019.88430. 10.5114/aoms.2019.88430 PMC7286316 32542089

[5] Summerton DJ, Kitrey ND (2012). Lumen N, Serafetinidis E, Djakovic N, European Association of Urology. EAU guidelines on iatrogenic trauma. Eur Urol.

[6] Cordon BH, Fracchia JA, Armenakas NA (2014). Iatrogenic nonendoscopic bladder injuries over 24 years: 127 cases at a single institution. Urology..

[7] Pereira BM, de Campos CC, Calderan TR (2013). Bladder injuries after external trauma: 20 years experience report in a population-based cross-sectional view. World J Urol.

[8] Figler BD, Hoffler CE, Reisman W, et al. Multi-disciplinary update on pelvic fracture associated bladder and urethral injuries [published correction appears in Injury. 2012;43:1242–1249. 10.1016/j.injury.2012.03.031 22592152

[9] Golan S, Baniel J, Lask D (2010). Transurethral resection of bladder tumour complicated by perforation requiring open surgical repair—clinical characteristics and oncological outcomes. BJU Int.

[10] El Hayek OR, Coelho RF, Dall’oglio MF (2009). Evaluation of the incidence of bladder perforation after transurethral bladder tumor resection in a residency setting. J Endourol.

[11] Collado A, Chechile GE, Salvador J, Vicente J (2000). Early complications of endoscopic treatment for superficial bladder tumors. J Urol.

[12] Gomez RG, Ceballos L, Coburn M (2004). Consensus statement on bladder injuries. BJU Int.

[13] Remmele W, Stegner HE (1987). Vorschlag zur einheitlichen Definition eines Immunreaktiven Score (IRS) für den immunhistochemischen Ostrogenrezeptor-Nachweis (ER-ICA) im Mammakarzinomgewebe [Recommendation for uniform definition of an immunoreactive score (IRS) for immunohistochemical estrogen receptor detection (ER-ICA) in breast cancer tissue]. Pathologe..

[14] Skolarikos A, Chrisofos M, Ferakis N, Papatsoris A, Dellis A, Deliveliotis C (2005). Does the management of bladder perforation during transurethral resection of superficial bladder tumors predispose to extravesical tumor recurrence?. J Urol.

[15] Mydlo JH, Weinstein R, Shah S, Solliday M, Macchia RJ (1999). Long-term consequences from bladder perforation and/or violation in the presence of transitional cell carcinoma: results of a small series and a review of the literature. J Urol.

[16] Zhang KY, Xing JC, Li W, Wu Z, Chen B, Bai DY (2017). A novel transurethral resection technique for superficial bladder tumor: retrograde en bloc resection. World J Surg Oncol.

[17] Bansal A, Sankhwar S, Goel A, Kumar M, Purkait B, Aeron R (2016). Grading of complications of transurethral resection of bladder tumor using Clavien-Dindo classification system. Indian J Urol.

[18] Gregg JR, McCormick B, Wang L (2016). Short term complications from transurethral resection of bladder tumor. Can J Urol.

[19] De Nunzio C, Franco G, Cindolo L (2014). Transuretral resection of the bladder (TURB): analysis of complications using a modified Clavien system in an Italian real life cohort. Eur J Surg Oncol.

[20] Pycha A, Lodde M, Lusuardi L (2003). Teaching transurethral resection of the bladder: still a challenge?. Urology..

[21] Balbay MD, Cimentepe E, Unsal A, Bayrak O, Koc A, Akbulut Z (2005). The actual incidence of bladder perforation following transurethral bladder surgery. J Urol.

[22] Poletajew S, Krajewski W, Kaczmarek K, et al. The learning curve for transurethral resection of bladder tumour: how many is enough to be independent, safe and effective surgeon? J Surg Educ. 2020. 10.1016/j.jsurg.2020.02.010. 10.1016/j.jsurg.2020.02.010 32147466

[23] Volikova AI, Marshall BJ, Yin JMA, Goodwin R, Chow PE, Wise MJ (2019). Structural, biomechanical and hemodynamic assessment of the bladder wall in healthy subjects. Res Rep Urol.

[24] Hakenberg OW, Linne C, Manseck A, Wirth MP (2000). Bladder wall thickness in normal adults and men with mild lower urinary tract symptoms and benign prostatic enlargement. Neurourol Urodyn.

[25] Serati M, Salvatore S, Cattoni E, Soligo M, Cromi A, Ghezzi F (2010). Ultrasound measurement of bladder wall thickness in different forms of detrusor overactivity. Int Urogynecol J.

[26] Morales-Orcajo E, Siebert T, Böl M (2018). Location-dependent correlation between tissue structure and the mechanical behaviour of the urinary bladder. Acta Biomater.

[27] Miodoński AJ, Litwin JA (1999). Microvascular architecture of the human urinary bladder wall: a corrosion casting study. Anat Rec.

